# Nerve Dependence in Colorectal Cancer

**DOI:** 10.3389/fcell.2022.766653

**Published:** 2022-02-10

**Authors:** Lincheng Zhang, Ludi Yang, Shuheng Jiang, Minhao Yu

**Affiliations:** ^1^ Department of Gastrointestinal Surgery, Renji Hospital, School of Medicine, Shanghai Jiao Tong University, Shanghai, China; ^2^ Department of Ophthalmology, Ninth People’s Hospital, Shanghai Jiao Tong University School of Medicine, Shanghai, China; ^3^ State Key Laboratory of Oncogenes and Related Genes, Shanghai Cancer Institute, Ren Ji Hospital, Shanghai Jiao Tong University School of Medicine, Shanghai, China

**Keywords:** colorectal cancer, tumor-nerve interaction, perineural invasion, mechanism, tumor microenvironment

## Abstract

Cancerous invasion of nerves has been reported in a list of malignant tumors as a high-risk pathological feature and marker of poor disease outcome especially in neurotrophic cancers (such as in pancreas and prostate), indicating that although once neglected, nerves could have played a pivotal role in tumorigenesis and cancer progression. In colorectal cancer, perineural invasion, a specific form of tumor-nerve interaction referring to the identification of tumor cells in proximity to the nerve, has been recognized as a strong and independent prognosis predictor; denervation of autonomic nerves and enteric nerves have shown that the existence of these nerves in the gut are accompanied by promoted cancer proliferation, further supporting that nerve is a potential accomplice to shield and nurture tumor cells. However, the precise role of nerve in CRC and the pattern of interaction between CRC cells and nerve has not been unveiled yet. Here we aim to review some basic knowledge of the importance of nerves in CRC and attempt to depict a mechanistic view of tumor-nerve interaction during CRC development.

## Background

Colorectal cancer (CRC) has been steadily ranked as one of the most common malignant tumors and a dominating cause of death in cancer patients. Although diverse pathogenic pathways have been identified in colorectal cancer, very few of them are targeted in clinical therapy.

It was not until previous decades that the significance of nerves in tumors received much attention. Recent studies have demonstrated that just as lymphovascular invasion, nerve involvement also lends support to tumor progression and dissemination, suggesting that neoplastic invasion of nerves might be another key hallmark of cancer. In clinical practice, perineural invasion (PNI), defined as at least 33% of nerve fiber circumference surrounded by cancer cells or cancer cells directly found in any layer of the nerve sheath (i.e. epineurium, perineurium or endoneurium) (Liebig et al., 2009), is most commonly used to evaluate the extent of nerve involvement. First described in head and neck cancer, the perineural invasion has now been reported in a list of malignant tumors including those in the pancreas, prostate, breast, colon and rectum, stomach, etc. Among all these cancers, despite the varying incidence of perineural invasion, PNI positive patients are remarkably susceptible to cancer progression and relapse, denoting novel pathways of tumorigenesis and metastasis to be discovered.

In colorectal cancer, perineural invasion occurs far less frequently than in pancreatic cancer, but once confirmed, it is responsible for rapid disease progression and remarkably unfavorable clinical outcomes. In most studies, detection rate of PNI in colorectal cancer stays less than 30%, suggesting a relatively low incidence of nerve involvement. *In-vitro* 3D migration assay also revealed that neurotrophic growth was far less frequent for colon and rectal cancer cells than pancreatic cancer cells (Liebl et al., 2013). However, these PNI-positive CRC patients suffered from severely compromised disease outcomes. As an independent risk factor of CRC prognosis, PNI culminates in more than 20% decrease in overall and disease-free survival. Several large-population studies further point out that PNI increases the risk of lymph node metastasis, another pathologic feature associated with poor disease prognosis, and cancer-related mortality. The latest studies on the association between PNI and disease prognosis are summarized in Table 1 (Leijssen et al., 2019; Skancke et al., 2019; Sun et al., 2019; Lee et al., 2020; Liu et al., 2020; Martínez Vila et al., 2020; Mo et al., 2020). Here we review the role of nerves in colorectal cancer, identify nerve dependence in colorectal cancer and illustrate underlying molecular mechanisms of perineural invasion.

**TABLE 1:** Significance of perineural invasion in predicting prognosis.

Cancer	Stage	Treatment	Incidence of PNI(%)	Clinical significance	Ref
CRC	T1-2,N0-2,M0	Surgical resection and/or chemotherapy	2.3	PNI is an independent high-risk factor of lymph node metastasis(LNM); lymph node metastasis rate is 40.7% in PNI-positive patients compared to 19.0% in PNI-negative patients	(Lee et al., 2020; Martínez Vila et al., 2020; Mo et al., 2020)
				PNI negatively influences DFS together with LNM (HR = 3.641, *p* < 0.001)	
colon	T1-T2	Surgical resection	3.4		
CRC	T1	Endoscopic resection	3.8		
CRC	Tis-T1N0M0	Mixed (surgery, endoscopy, chemotherapy, radiotherapy)	11.1	PNI is among one of the predictors in the survival nomogram to predict 1-year, 3-years and 5-years OS	Liu et al. (2020)
Rectum	locally advanced(T3/T4, N+)	Surgical resection with/without neoadjuvant chemoradiotherapy	24.3	3-years DFS rate is 76.8% in PNI-negative patients compared to 26.2% in PNI-positive patient	Sun et al. (2019)
				3-years OS rate is 82.8% in PNI-negative patients compared to 31.0% in PNI positive patients(*p* < 0.001)	
Colon	II	Surgical resection with/without adjuvant chemoradiotherapy	3.8	PNI attributes to 32.1% increase of 5-years mortality	Skancke et al. (2019)
Colon	I-III	Surgical resection with/without adjuvant chemotherapy	18.8	5-years DFS is 85.4% in PNI-negative patients compared to 57.8% in PNI-positive patients(*p* < 0.001)	Leijssen et al. (2019)
				5-years OS rate is 76.6% in PNI-negative patients compared to 53.2% in PNI positive patients(*p* < 0.001)	
				PNI is associated with higher risk of disease recurrence and cancer death(*p* < 0.001)	

DFS, disease-free survival; OS, overall survival; HR, hazard ratio; MMR, mismatched repair defects; DSS, disease-specific survival.

## Nerves in Colorectal Cancer

Colon and rectum are richly innervated by autonomic nerves and enteric nerves--presumably deal for cancer cells to take advantage of--yet the precise role of nerve in colorectal cancer has not been delineated. Colorectal distribution of nerves can be categorized into two groups: extrinsic innervation incorporates sympathetic and parasympathetic input from the brain and spinal cord, while intrinsic innervation refers to the enteric nervous system, which integrates signals from autonomic input and controls gut secretion, reabsorption and motility. Ascending and transverse colon receives sympathetic innervation through superior mesenteric plexus and parasympathetic innervation through vagus nerve; descending colon and upper rectum receives sympathetic innervation *via* inferior mesenteric plexus, lower rectum *via* inferior hypogastric plexus, with common parasympathetic input through the pelvic splanchnic nerve. Both colon and rectum are regulated by the enteric nervous system (ENS), namely the myenteric (Auerbach’s) and submucosal (Meissner) plexus. Together these nerves orchestrate the normal functioning of gut **(** Table 2 **)**.

**TABLE 2:** Physiological innervation of nerves in CRC and denervation studies.

	Nerve innervation	Location				Method of denervation	Effect of denervation
		Ascending colon	Descending colon	Upper rectum	Lower rectum		
Extrinsic	Sympathetic	Thoracic splanchnic nerve (superior mesenteric plexus)	Lumbar splanchnic nerve(inferior mesenteric plexus)	Lumbar splanchnic nerve(inferior mesenteric plexus)	Sacral splanchnic nerve(inferior hypogastric plexus)	Beta receptor blockade;	↓cancer cell proliferation and survival *in vitro*; Probably improves CRC patients’ clinical outcome
	Sensory			Pelvic splanchnic nerve		/	Unclear
	Parasympathetic	Vagus nerve	Pelvic splanchnic nerve			M3R blockade	↓cancer cell proliferation, tumor number and size in vitro and in vivo.
Intrinsic	Myenteric plexus					Chagasic megacolon, BAC treatment;	↓preneoplastic and neoplastic lesions
							↓risk of developing colon cancer
	Submucosal plexus					/	Unclear

### Histopathological Changes of Nerves in CRC

Structural disarrangement of nerve tissues in CRC patients has been reported by several studies, indicating putative roles of autonomic and enteric nerves in the development of CRC. A retrospective study of 90 CRC patients tested the immunoreactivity of tyrosine hydroxylase and vesicular acetylcholine transporter respectively in the resected tissue to track down sympathetic and parasympathetic nerves. Results denoted the existence of both sympathetic and parasympathetic nerves in proximity to the tumor. Sympathetic nerves are found in the stroma closer to tumor site, while parasympathetic nerves are located away from tumor cells (Zhou et al., 2018). Distribution of β2A receptors appears to be denser in CRC-invaded ganglia and larger nerve bundles, compared to the nervous tissue in non-tumor sections (11). Besides, an increasingly diffuse and dense expression pattern of β2A receptors is observed from the normal colon tissue to G1, G2 and G3 differentiated adenocarcinoma. There is also a significant association between *β2* adrenergic receptor expression and tumor size, tumor invasion or lymph node metastasis. Several studies have reported abnormal morphology of enteric nerves. Significant loss of myenteric and submucosal plexuses was noticed in CRC, especially in more progressive types (Godlewski, 2010; Ciurea et al., 2017); in sigmoid and rectal cancer, disruption of normal ENS structures are observed, with an irregular border of the myenteric plexus and deformation of their structures, and, simultaneously, a larger area of extracellular matrix surrounding the myenteric plexuses with more abundant collagen fibers in tumor-infiltrated colon walls (Zauszkiewicz-Pawlak et al., 2017). Myelin-like structures that usually appear in degeneration of nerves are observed near the tumor invasion site. A marked drop in the density of enteric glial cells was also observed corresponding to the increase in tumor grading (Jaiswal et al., 2020).

### Denervation Studies

Sustained efforts have been made in denervation studies to ascertain whether loss of nerve innervation would affect tumor initiation and survival, providing functional insights into the role of nerves in CRC.

Research of parasympathetic denervation mainly considers ablation of muscarinic receptors. Targeting muscarinic acetylcholine receptor 3 (M3R) through selective and non-selective antagonists (*p*-fluorohexahydro-sila-difenidol hydrochloride and atropine respectively) repressed H508 colon cancer cell proliferation by approximately 40% whilst acetylcholinesterase inhibitors were capable of stimulating tumor growth by 2 to 2.5 fold (Cheng et al., 2008), indicating participation of cholinergic signaling in tumor development. Similar inhibitory effects were also observed in murine colon cancer models as epithelial proliferation, tumor size and quantity were greatly diminished in mice deficient of CHRM3, the coding gene of M3R (Raufman et al., 2008).

Other denervation studies have been focused on enteric nerve denervation. Chagas disease, a trypanosomiasis-related disease that induces megacolon and damage to myenteric neurons, seems to be an existing example of how myenteric neuronal activity negatively associates with colon carcinogenesis. Experiments on Wistar rats suggested that chronic infection with Trypanosoma cruzi led to fewer tumors when treated with dimethylhydrazine(DMH), a specific chemical carcinogen to induce CRC (Oliveira et al., 2001). A review of histopathological findings from 894 chagasic megacolon patients revealed that none of those patients developed colon cancer(Garcia et al., 2003); previous studies on surgical specimens from Chagas patients who underwent megacolon resection confirmed a decline in the density of myenteric neurons and correspondingly reduced risk for colon cancer [(Kannen et al., 2015)]. To prove the tumorigenic effect of myenteric nerves, *in-vivo* experimental approach has been established to mimic myenteric denervation in chagasic megacolon through benzalkonium chloride (BAC) treatment. Results showed that BAC-treated rats are less susceptible to DMH, since both preneoplastic and neoplastic lesions were reduced compared to those whose myenteric innervation were intact (Garcia et al., 1996; Vespúcio et al., 2008; Kannen et al., 2015).

Although few studies have directly investigated changes in tumors upon sympathetic denervation, many researchers have reported effects of beta receptor antagonists. Studies of multiple CRC cell lines demonstrated that beta blockade inhibits cell viability and proliferation in a dose-dependent manner, probably through EGFR-Akt/ERK1/2 pathway, cell cycle arrest and apoptosis followed by suppression of β2 signaling (Coelho et al., 2015; Chin et al., 2016). Meanwhile, various clinical studies were conducted in Europe to measure the effect of beta receptor blockers on CRC patient outcomes, but no definitive conclusion has been made. Stage-specific benefit of beta blockade at diagnosis (particularly selective beta blockers) was once detected, as overall and CRC-specific mortality markedly decreased in stage IV patients prescribed with beta blockers (Jansen et al., 2014). However, several large population-based studies have reported that long-term beta blocker usage may contribute to increased risk of CRC progression, and little association was found between pre- or post-diagnostic beta blockade and improved clinical outcomes (Jansen et al., 2012; Hicks et al., 2013; Jansen et al., 2017). Perioperative application of beta blockers, on the other hand, seems to be more promising in improving CRC patients’ prognosis. The latest study demonstrated that preoperative beta blocker treatment results in reduced post-operative complications and mortality in rectal cancer (Ahl et al., 2020); it was also previously found that combine perioperative blockade of *β* receptor and COX2 leads to lower recurrence rate and improvement in tumor biomarkers associated with epithelial-to-mesenchymal transition, immune microenvironment and CRC-related inflammatory pathways (Haldar et al., 2020).

## Underlying Mechanisms of Perineural Invasion

Initially, cancer cells were thought to spread along the nerves passively because perineural connective tissues are loose paths with low resistance. With a distensible space between nerves and outer sheaths, tumor cells might be capable of lurk in those spaces without showing any symptoms. Later, however, ultrastructural studies revealed the presence of densely organized collagen and basement membrane constituting the nerve sheath, which should have highly resisted the invasion of cancer cells, indicating that tumors could take the initiative to disseminate through nerves (Liebig et al., 2009). Through concerted efforts of neurotrophic factors, neurotransmitters, adhesion molecules, matrix metalloproteinases, glial cells and tumor stem cells, a perineural niche favoring cancerous infiltration is eventually established, and neoplastic cells are guided towards this route.

### Neurotrophic Factors

Neurotrophic factors represent a list of proteins binding to tyrosine kinase receptors to activate downstream signaling pathways in the growth and differentiation of nerves, comprising nerve growth factor (NGF), brain-derived neurotrophic factor (BDNF), glial cell-derived neurotrophic factor (GDNF), neurotrophin-3 (NT-3) and neurotrophin-4/5 (NT-4/5). Their high-affinity receptors include tropomyosin-related kinase (Trk), preferring NGF, BDNF and NT-3/4, while GDNF family receptor (GFRα)/RET prefers GDNF. Each ligand-receptor complex, leading to phosphorylation of various downstream pathways including PI3K/Akt and Ras/MAPK, is responsible for recruitment of specific type of nerves during nerve development. NGF/TrkA signaling dominates in the establishment of sympathetic innervation, GDNF/GFRα in parasympathetic innervation and BDNF/TrkB in sensory innervation. Multifaceted interactions between epithelium and neurons in embryonic establishment of glandular innervation are summarized by Zahalka et al. (Zahalka and Frenette, 2020). In physiological conditions, epithelial mesenchyme secrets neurotrophins to bind their specific receptors, forming a ligand-receptor complex to be engulfed by axons and retrogradely transported to the soma, thus stimulating down-stream transcription, axonogenesis, and nerve recruitment to the target organ. Once morphogenesis of their target organ is accomplished, neurotrophic factors cease to accumulate (Klein, 1994).

However, these molecules indicated in embryonic development of nervous system can be re-stimulated in the context of cancer. Neurotrophic factors such as NGF and GDNF can be released either by tumor cells or neurons (Amit et al., 2016). In colorectal cancer, abnormality in TrkA has been recurrently reported. Chromosomal rearrangement of TrkA coding gene NTRK1 produces NTRK1 fusion proteins and upregulates TrkA kinase activity, followed by oncogenic phosphorylation of downstream proteins and hypersensitivity to NGF binding. TPM3-NTRK1 is the most commonly identified fusion gene, as TrkA itself was originally separated from TPM3-TrkA fusion gene of colon carcinoma, with a detection rate of 0.5–1%. Other fusion forms such as TPR-NTRK1 and LMNA-NTRK1 have also been reported. Indeed, TrkA positive patients may represent a minority of patients with satiable response to TrkA kinase inhibitors. In resected colon adenocarcinoma, cytoplasmic immunoreactivity to TrkA was abundantly detected (Ardini et al., 2014). *In vitro* experiments confirmed the presence of typical TPM3-NTRK1 fusion gene in KM12 human CRC cell line and demonstrated strong effects of TrkA inhibitor on suppressing cancer cell proliferation. TrkA fusion protein was also discovered in a case of liver metastasis of CRC, and application of Entrectinib, a selective Trk inhibitor, managed to achieve clinical partial response (Sartore-Bianchi et al., 2016).

Other neurotrophic factors such as brain-derived neurotrophic factor (BDNF) and glial cell-derived neurotrophic factor (GDNF), have been shown to induce tumor migration *via* upregulation of VEGF and activation of p38 and PI3K/Akt signaling pathway (Huang et al., 2014; Huang et al., 2015). *In-vitro* studies in two human colon cancer cell lines, HCT116 and SW480, proved that migratory activity increases with the concentration of BDNF and GDNF respectively, paralleled with increased expression of VEGF, MAPK and PI3K/Akt-associated proteins at transcription and translation level.

So far, it has not been fully revealed in CRC how neurotrophic factors get engaged in perineural invasion(PNI), but accumulating evidence in other types of cancer indicates crucial importance of neurotrophin signaling through the development of nerve invasion. In pancreatic cancer, GDNF signaling is a characterized mechanism of PNI (Gil et al., 2010; He et al., 2014). *In-vitro* co-culture of dorsal root ganglia and human pancreatic adenocarcinoma cell lines and *in-vivo* murine sciatic nerve models of PNI showed that nerves release GDNF, and induce polarized neurotrophic migration of cancer cells *via* downstream pathways of RET. Tumor invasion of nerves is upregulated upon GDNF induction and is greatly diminished when GDNF/GFRa1/RET pathway is suppressed. Similar changes have been reported in prostate cancer, as GFRα-1/RET binding of GDNF potentiates invasion and proliferation of cancer cells (Ban et al., 2017). NGF and GDNF were also found to be more abundantly expressed in patients with PNI and higher Gleason scores (Baspinar et al., 2017). In salivary adenoid cystic carcinoma (SACC), NT-3/TrkC signaling enhances *in-vitro* cancer cell migration in the presence of Schwann cells: A comparative study of 78 SACC and 25 normal tissue specimens demonstrated higher expression of NT-3 in tumor cells surrounding the nerves and TrkC in tumor-invaded nerves; statistics proved that level of NT-3/TrkC expression is strongly correlated with the occurrence of PNI (Li et al., 2019). BDNF/TrkB-dependent Schwann-like differentiation was also discovered in human SACC cell lines; both TrkB and S100A4, a surface marker of Schwann cell, are found to be significantly associated with PNI (Jia et al., 2015; Shan et al., 2016).

It can be concluded that neurotrophins have multifaceted roles in tumor-nerve crosstalk of PNI, as both tumor cells and neurons are able to release neurotrophic factors. These growth factors might be secreted by neoplastic epithelium in the same way as embryonic neurogenesis to summon newborn nerves to the invasive front, facilitating direct contact between cancer cells and nerves; or they might be secreted by nerves to polarize neurotrophic migration of cancer cells towards resident nerves, as indicated in pancreatic cancer, prostate cancer, SACC, etc. Further research is needed in CRC to unveil the role of neurotrophic factors in bridging tumor and nerve.

### Neurotransmitters

As the main effector molecules released by nerve fibers, neurotransmitters may serve as a crucial messenger between tumor and nerve. Upregulated adrenergic signaling has been reported in colorectal cancer. As indicated in several pathology reports of CRC tissue, distribution of beta receptors appears to be denser in the tumor site (Godlewski, 2010; Ciurea et al., 2017), and such phenomenon is usually accompanied by worse prognosis. Some studies further demonstrate a direct relationship between adrenergic transmitters and tumor proliferation in CRC. An *in-vitro* study of Wong et al. shows COX-2 dependent stimulation of adrenaline on human colon adenocarcinoma HT-29 cell proliferation (Wong et al., 2011), as adrenaline activates COX-2, VEGF, PGE2 and MMP-9 downstream, which can be rescued by COX-2 inhibitors and beta receptor antagonists. Besides, adrenaline-induced COX-2 upregulation has been found to suppress immunity in coordination with inflammatory signals by enhancing expression of IDO and IL-10 in macrophages, thus attenuating the proliferation and IFN-γ production of CD8^+^ T cells and facilitating immune escape in colon cancer (Muthuswamy et al., 2017). Han et al. have revealed the role of epinephrine in colorectal cancer as well. By targeting the CREB1-miR-373 axis and subsequent downregulation of tumor suppressor gene TIMP2 and APC, epinephrine plays a stimulatory role in the proliferation and dissemination of human colon cancer cells *in vitro* and *in vivo* (Han et al., 2020).

Cholinergic signaling via acetylcholine (Ach) and its receptors constitutes another regulating pathway of tumor-nerve crosstalk. Mounting evidence has shown that muscarinic receptor participates in the development of CRC. Muscarinic acetylcholine receptor 1 (M1R) and 3 (M3R) are the predominant types distributed in the gut, through which enteric and autonomic nervous system innervates intestinal smooth muscle tone, with M3R notably over-expressed in CRC lesions. Expression of CHRM3, coding gene of M3R robustly increased by 128 fold in colon adenocarcinoma compared with that in adjacent normal epithelium; immunostaining also shows over-expression of M3R in cancerous tissue in contrast to the unaffected epithelium (Cheng et al., 2017). CHRM3-deficient mice develop fewer and smaller colon neoplasm induced by Azoxymethane, whereas CHRM1 knockout or dual knockout didn’t have much visible impact on tumor size or quantity (Cheng et al., 2014).

Due to ubiquitous distribution and multi-target effect of adrenergic and cholinergic transmitters, it would be troublesome to distinguish whether neurotransmitters support cancer development through direct local interaction with tumor or via systemic neuroendocrine signaling activated by other CRC-related events. Allen et al. have identified a feed-forward loop in ovarian cancer cells, that in response to sustained adrenergic signaling, tumor cells secrete BDNF, which then promotes intratumoral innervation via host neurotrophic receptor tyrosine kinase 2 (TrkB) receptors (Allen et al., 2018), denoting that sympathetic pathway might be an upstream inducer of tumor innervation. Parasympathetic signaling through acetylcholine promotes matrix metalloproteinase 1 (MMP1) expression, facilitating the invasion of HT29 and H508 human colon cancer cells, which can be rescued by atropine and anti-MMP1 antibody (Raufman et al., 2011). Potential mechanistic pathway downstream of M3R activation was reviewed in detail by Ali et al. (Ali et al., 2021), which involves stimulation of protein kinase C-α (PKCα), MMP1 and MMP7 transcription, EGFR signaling and PI3K/Akt pathway and ultimately contributes to tumor growth, dissemination and invasion. Thus, neurotransmitters and their receptors might work as a stimulator to trigger invasive acts of CRC cells, yet more studies are required to identify the role of autonomic signaling in tumor-nerve interaction.

### Adhesion Molecules

During the maturation of the nervous system, a wide range of cell-matrix and cell-cell interactions are required to facilitate neuronal migration, axon/dendrite outgrowth and synaptogenesis, mainly mediated by the immunoglobulin superfamily of neural cell adhesion molecules (CAMs). L1CAM is a crucial family of adhesion molecules comprising L1 and its close homolog (CHL1), NrCAM and neurofascin. Their extracellular domain consisted of immunoglobulin (Ig)-like and fibronectin type III-homologous (FnIII) domains, specializes in modulating intercellular interaction, particularly at synaptic cleft to assist in synapse forming and functioning. L1 also stimulates signal transduction related to neuron migration and neurite outgrowth, either by serving as signaling molecule itself or as co-receptor(Schmid and Maness, 2008; Sytnyk et al., 2017). By interacting with *β1* integrin, L1 helps to upregulate cell-matrix adhesion and initiates downstream cascade, resulting in ERK1/2 MAPK signaling and transcriptional activation of a series of proteins that upregulates cell motility, including integrin and Rac1.

A handful of researchers have proposed that L1CAM represents a potential marker for cancer invasiveness and progression of CRC. It was recurrently reported that L1 expression was correlated with increased tendency of metastasis, along with advanced cancer stage, high-risk pathological features (including poor differentiation grade, solid cancer nests and tumor budding) and severely shortened survival (Boo et al., 2007; Kaifi et al., 2007; Tampakis et al., 2020; Tieng et al., 2020). Transcription of L1CAM mRNA was significantly active in the invasive front compared to the center of tumor especially in patients with nodal involvement; besides, expression levels of L1CAM and ERK1/2 in CRC tissue is associated with lymph node metastasis, indicating supportive role of L1CAM-associated in CRC dissemination (Kajiwara et al., 2011; Fang et al., 2020). Regretfully, perineural invasion status was absent in most of these studies, making it difficult to identify whether L1 also helps tumor cells to infiltrate the nerves. Recently, it has been reported that colorectal neoplastic cells preferentially adhere to enteric neurons, supporting further dissemination of tumor cells. Through tumor-nerve adhesion mediated by L1CAM and N-Cadherin, CRC tumor cells can migrate along enteric neurons *in vitro*, representing a possible route for CRC perineural invasion due to the wide distribution of enteric nerves throughout the colon and rectum (Duchalais et al., 2018).

In pancreatic cancer, there is a strong association between L1CAM expression in cancer tissue and perineural invasion (Ben et al., 2010). By constructing *in-vitro* model of PNI, it was revealed that L1CAM secreted by Schwann cells attracts cancer cells into perineural niche via MAPK pathway and enhances expression of metalloproteinase (MMP) in cancer cells to facilitate penetration of extracellular matrix (Na’ara et al., 2019). Such process proves to be L1-dependent as neural invasion is greatly impaired after treatment of L1CAM antibody. Considering dual identity of L1CAM in nerve development and neoplastic invasion, more work remains to be done to pinpoint the position of L1CAM in neural invasion of CRC.

## Concluding Remarks

**FIGURE 1:**
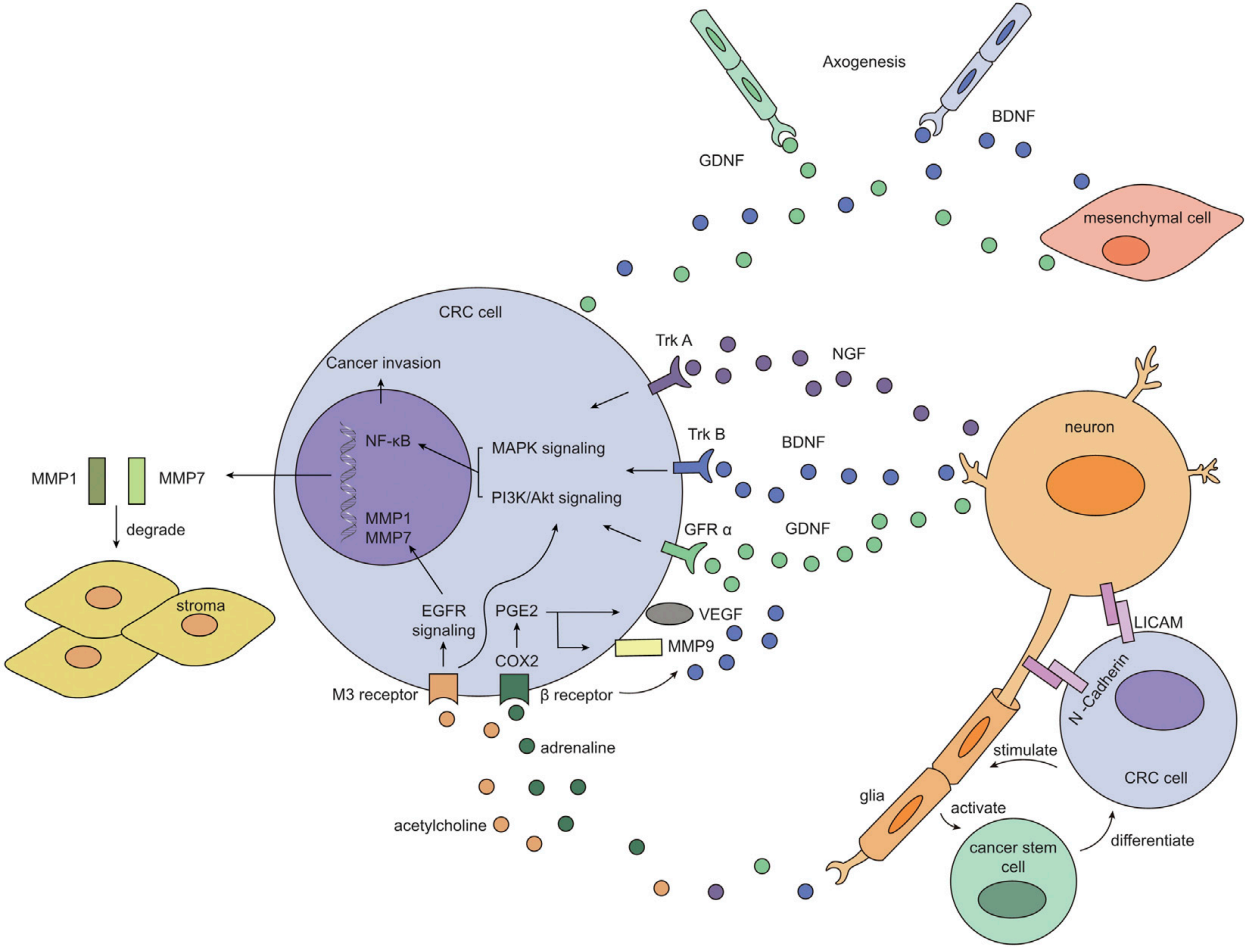
Possible pattern of interaction between CRC cell and adjacent nerve. Neurons secret neurotrophic factors such as NGF, BDNF and GDNF, which then form signaling complex with their cognate receptors (TrkA, TrkB and GFRα respectively) on CRC cells. These complexes initiate intracellular MAPK and PI3K/Akt pathway and eventually turn on NF-κB transcription, facilitating tumor survival and invasion. Besides, neurotransmitters released by neurons also switch on growth pathways in CRC cells and prompts extracellular matrix remodeling: adrenaline binds to *β* receptor and activates PGE2/COX2 axis, resulting in increased expression of VEGF and MMP9; acetylcholine binds to M3 receptor, which upregulates EGFR signaling and downstream production of MMP1 and MMP7. 2) Tumor cells themselves and mesenchymal cells may also release BDNF and GDNF to bind TrkB and GFRα expressed on newborn nerves, assisting in neurogenesis in the tumor microenvironment, thus “placing” nerves in proximity to tumor cells. Adhesion molecules such as L1CAM and N-Cadherin are expressed on CRC cells and neurons, allowing for further migration and dissemination of cancer cells along the adjacent nerve. 3) Glia cells may be stimulated by CRC cells to activate cancer stem cell, which serves as a replenishing pool for CRC cells, constituting a positive feedback loop.

Nerve dependence has been identified in several neurotrophic cancers, such as pancreatic cancer and prostate cancer, with numerous studies focused on deciphering potential mechanisms of perineural invasion. In contrast, the role of nerves in CRC has received far less attention, possibly due to low incidence of PNI in CRC (less than 30%). Currently, established models for neural invasion (Amit et al., 2016) (such as *in-vitro* dorsal root ganglia assay, 3D Schwann cell outgrowth and migration essay, etc.) are rarely applied in CRC-related studies, making it difficult to pattern nerve involvement in a systematic approach. Although the pattern of direct interaction between cancer cells and nerves has not been clearly elucidated, a handful of clues have suggested that a specialized microenvironment might be established surrounding cancer cells and adjacent nerves, with neurotrophins, neurotransmitters, adhesion molecules, matrix metalloproteinase and other mediators highly enriched in this niche **(** Figure 1 **)**. It was also revealed that enteric glial cells, component of the neural sheath, might be activated by tumor cells to assist in expansion of cancer stem cells (Valès et al., 2019). As indicated in other types of cancers, neurotrophins may be secreted by neurons, mesenchyme or tumor cells, contributing to neurogenesis and further tumor survival or invasion; Adhesion molecules may serve as the “glue” between cancer cell and nerve to potentiate neural invasion; MMPs released by tumor cells help to degrade extracellular connective tissues, facilitating protrusion into the neural sheath; chemotaxis recruit other components and establish a favorable niche for neural tracking. Nerves may also advocate tumor growth by releasing neurotransmitters and activating multiple downstream pathways such as MAPK and PI3K/Akt signaling. Here we have illustrated the clinical significance of perineural invasion, a specific form of tumor-nerve, and we have exhibited a possible mechanistic view of nerve participation in CRC. Further studies need to be carried out to focus directly on local interaction between cancer cells and neurons or glial cells to provide solid evidence of nerve dependence in CRC.
